# Supplementary material for: Single-cell imaging of α and β cell metabolic response to glucose in living human Langerhans islets
Source: Commun Biol. 2022 Nov 12;5:1232. doi: 10.1038/s42003-022-04215-w (PMC9653440; doi:10.1038/s42003-022-04215-w)
Supplement: Supplementary file 2 — Supplementary Information [file 42003_2022_4215_MOESM2_ESM.pdf]

Supplementary figures and tables

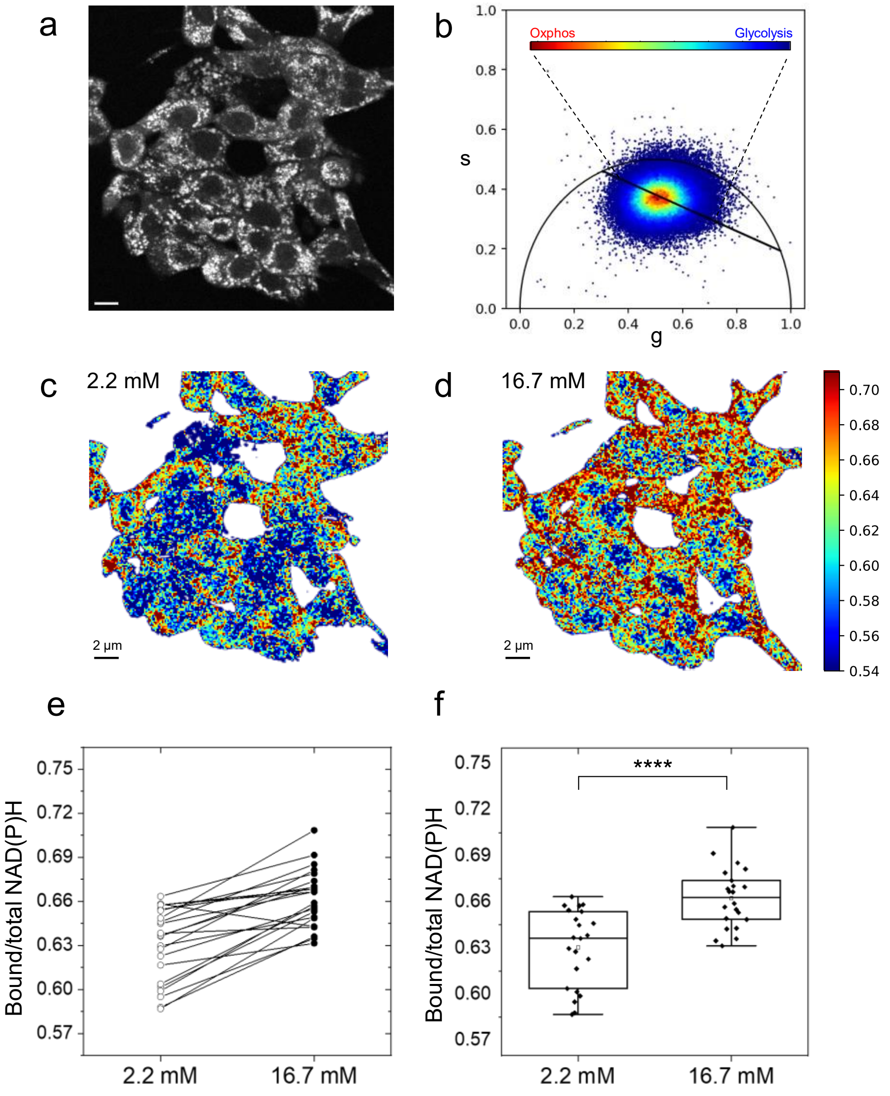

Fig. S1: Metabolic imaging of INS-1E cells. Before the FLIM acquisition, INS-1E cells were placed in starvation using SAB buffer supplemented with 2.2 mM glucose for 45 minutes, then imaged in the same conditions (**a**). As shown in (**b**), each pixel in the fluorescence-intensity image has a corresponding point in the phasor plot. Glucose stimulation was performed by adding glucose within the SAB buffer to reach a final concentration of 16.7 mM. After 3 minutes, the same cell-clusters acquired at low glucose were acquired upon glucose stimulation. (**c-d**) Color-coded FLIM maps at 2.2 mM and 16.7 mM were prepared to better appreciate the metabolic shift induced by glucose stimulation: blueish pixels present a shift towards the NAD(P)H free form compared to the median (green), while reddish are towards bound NAD(P)H. By comparing the two metabolic maps a clear whole-frame shift towards bound NAD(P)H can be observed. (**e**) The difference between 2.2

mM to 16.7 mM conditions determines the single-cell metabolic shift**.** (**f**) The average metabolic response (55 cells from n=3 independent samples) consists in a marked shift towards bound form (i.e. oxidative phosphorylation), in keeping with literature. Data reported and mean ±SD (Standard Deviation). Statistical difference obtained using Paired Samples Wilcoxon Test, with significance $\alpha = 0.05$ (*: $P < 0.05$; **: $P < 0.01$; ***: $P< 0.001$; ****: $P < 0.0001$). Scale bar: 2 μm.

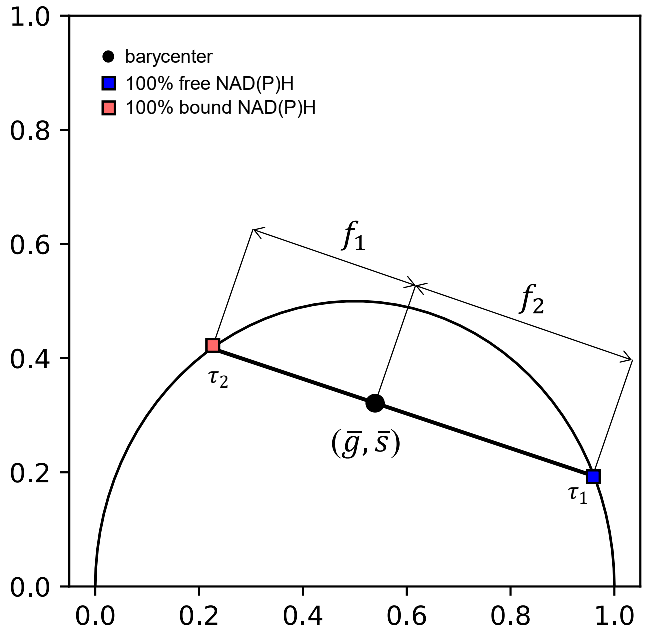

Fig. S2: Determination of Bound/total NAD(P)H ratio. Barycenter is calculated using the mathematical definition in **Eq. 2** and lies on the metabolic axis, i.e. the segment connecting the reference points at τ_1=0.4 ns (free NAD(P)H) and τ_2=3.4 ns (bound NAD(P)H). The barycenter coordinates can be converted into the Bound/total NAD(P)H ratio by **Eq. 3** in Materials and Methods. By definition, such ratio quantifies the fractional intensity of NAD(P)H in the 'bound' form with respect to the total NAD(P)H (bound + free) present in cell. Additionally, by definition, f_2=0 means that the phasor barycenter coincides with point τ_1, then NAD(P)H is exclusively in the free form, whereas f_1=0 means that the phasor barycenter coincides with τ_2 and then that NAD(P)H is exclusively in the bound form. The fractional intensities measured by FLIM can be converted into the actual (molar) fractions of 'bound' and 'free' NAD(P)H only by means of the quantum yields of the two species (see main text for further details).

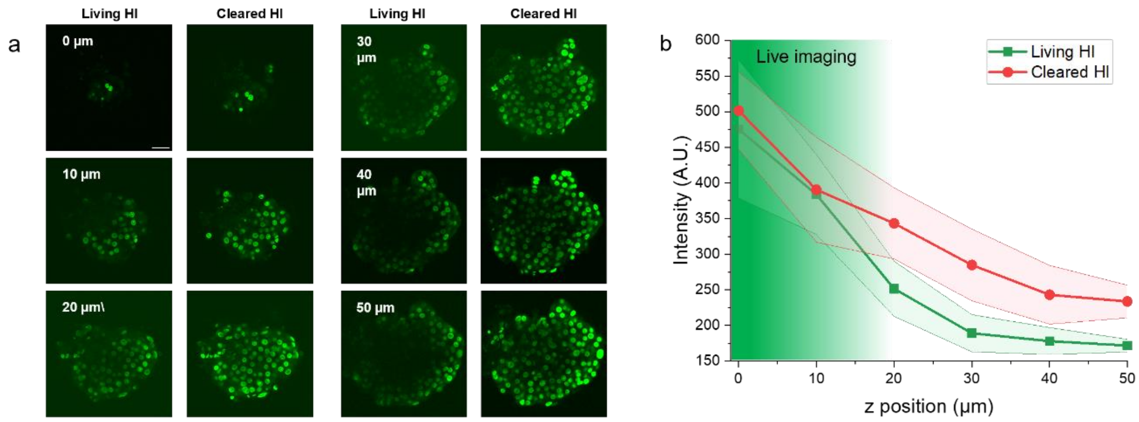

Fig. S3. Assessment of the optimal focal plane. (**a**) A living human islet (HI) has been imaged at different focal planes, starting from the ‘basal’ one and then progressively deeper within the islet (left columns). Then, the same islet was fixed and cleared to decrease the signal loss and finally imaged following the same procedure (right columns). (**b**) For each acquired image, the mean intensity value in the center of the image was derived and displayed in the plot. It can be observed that imaging in a living islet is acceptable only within the first 10-15 µm from the bottom (<20% loss of signal, approximately). By contrast, the use of clearing procedures allows acceptable imaging performances deeper within the tissue imaging. Scale bar: 30 µm.

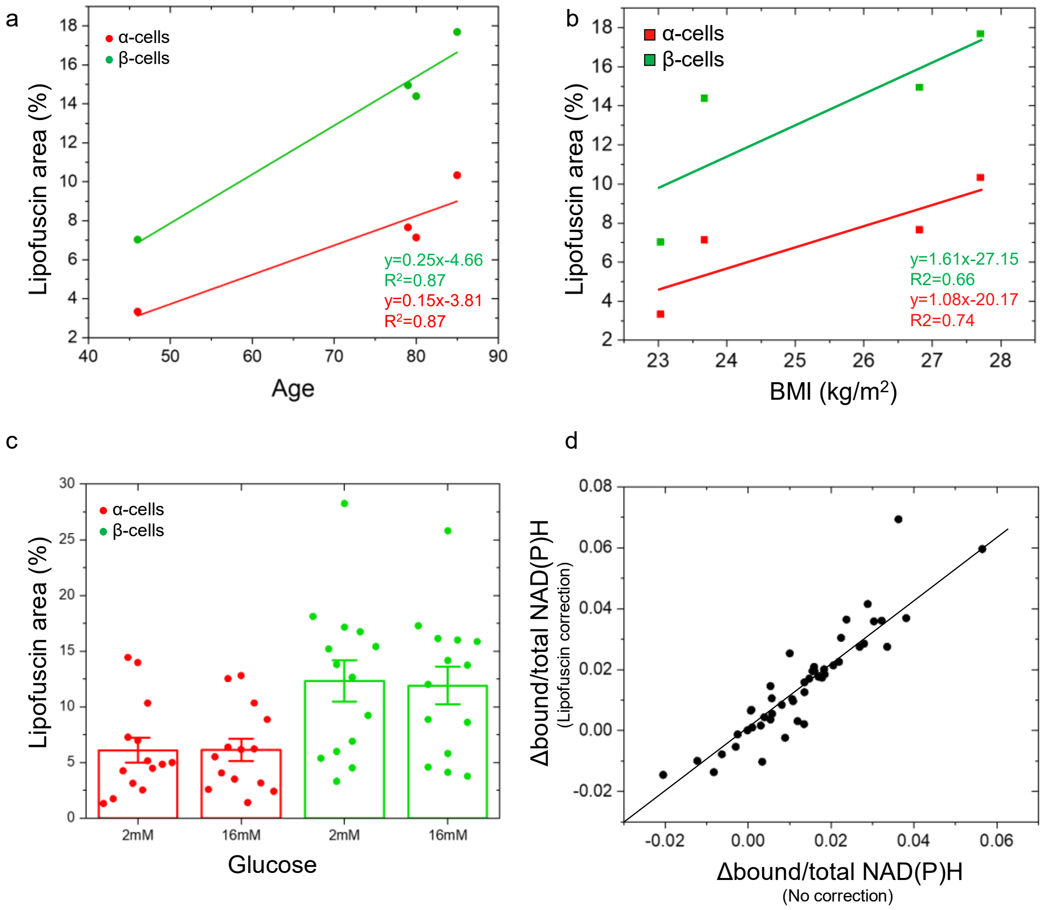

Fig. S4: Lipofuscin content among different donors and effects of glucose stimulation. (**a**) Lipofuscin content linearly correlates with donor age for both α- and β-cell types. The graph legend displays the fitting equation with the associated R^2 coefficient. (**b**) Lipofuscin content correlates with Body Mass Index for both α- and β-cells. The graph legend displays the fitting equation with the associated R^2 coefficient. **(c)** Glucose stimulation has no effect on lipofuscin content in both cell types. Data presented as donor Mean ± Standard Error (SE) values from n=14 islets. **(d)** To assess the effect of lipofuscin correction on metabolic shifts, the same cells were analyzed with and without lipofuscin correction. Most of the experimental points lie close to bisection line, showing that lipofuscin correction has a negligible effect on the derived metabolic shift. Each point in the graph represents a single cell in a representative islet.

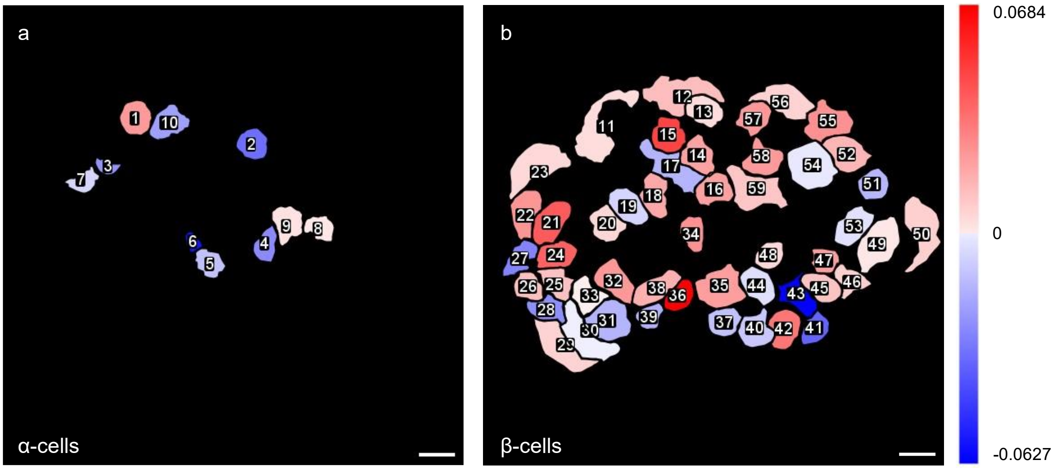

Fig. S5. Metabolic map of the representative islet in Fig. 4. Color-map encoding the magnitude of single-cell metabolic responses for α cells (**a**) and β cells (**b**). Metabolic shifts towards free NAD(P)H are reddish, those towards bound form are blueish, with magnitude encoded by color intensity.

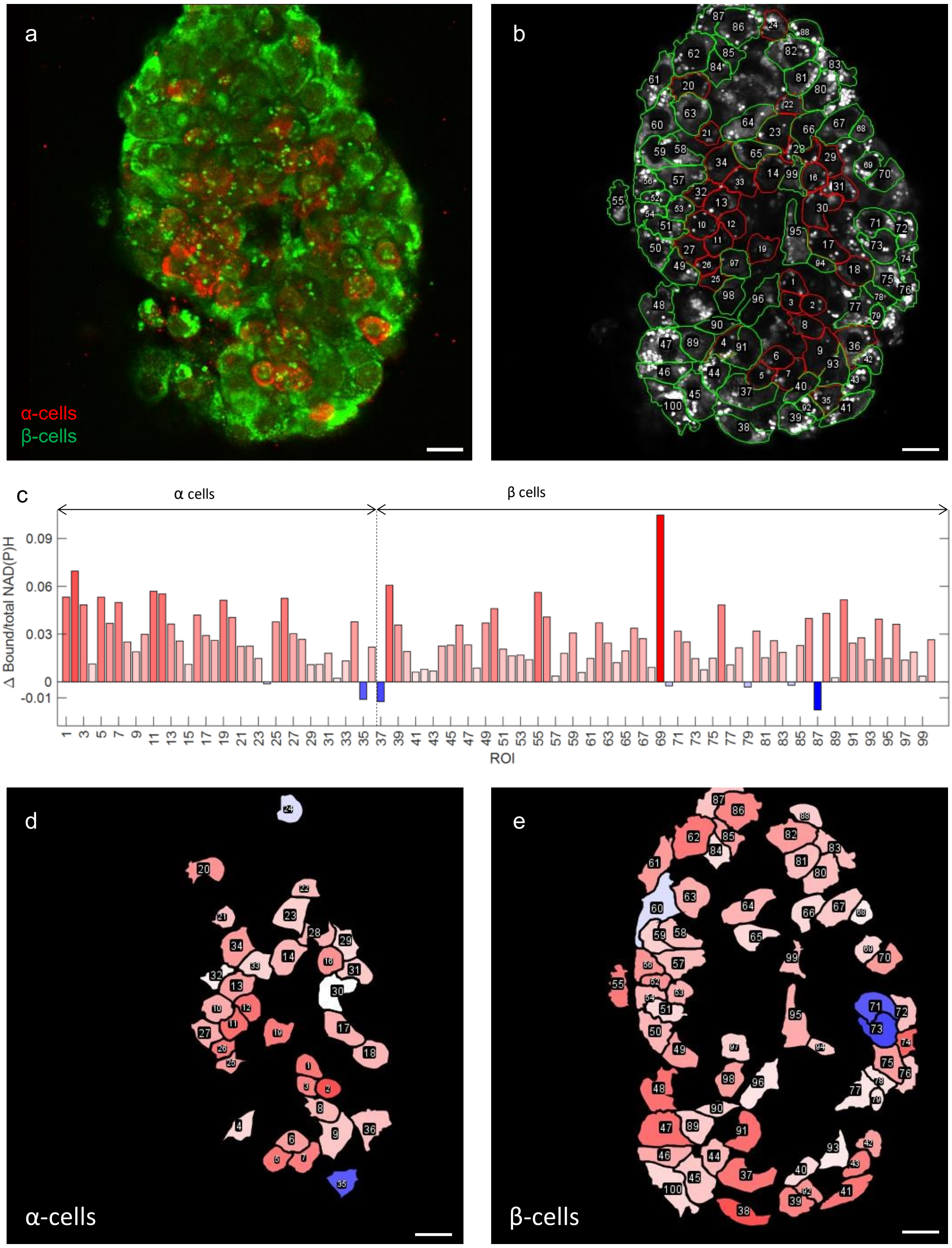

Fig. S6. Single-cell metabolic response of representative islet. (**a**) Post-fixation immunofluorescence allows discriminating α and β cells in the islet; (**b**) single-cell segmentation is performed. A unique identifier is assigned to each ROI to distinguish the same cell in both 2.2 mM and 16.7 mM conditions; (**c**) phasor-FLIM barycenter displacement along the metabolic axis upon glucose stimulation determines the metabolic shift (Δbound/total NAD(P)H) displayed here; (**d-e**) Color-map encoding the magnitude of single-cell metabolic responses for α cells (**d**) and β cells (**e**).

Metabolic shifts towards free NAD(P)H are reddish, those towards bound form are blueish, with magnitude encoded by color intensity.

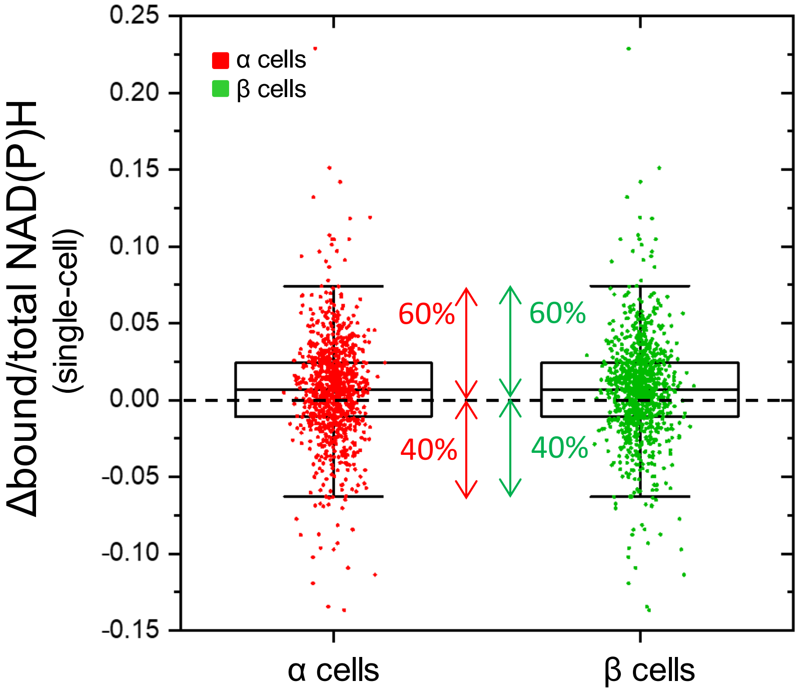

Fig. S7. Single-cell metabolic shift response. Heterogeneity of response can be found in both α- (n_α=312 cells collected) and β-cells (n_β=654 cells collected): in both cell types, approximately 60% of cells display a metabolic shift towards higher bound/total NAD(P)H values (and the remaining 40% towards lower values).

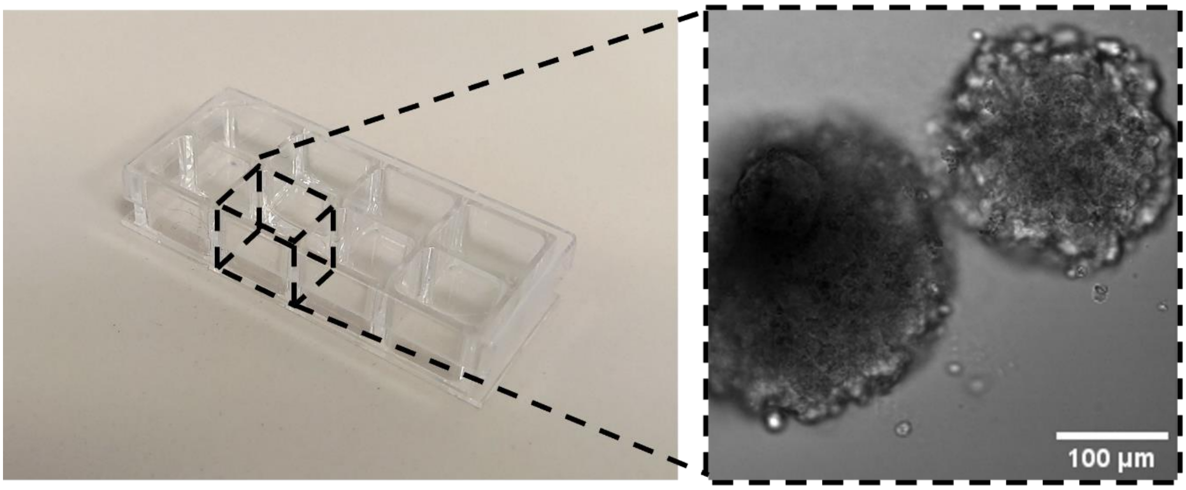

Fig. S8 Human islets culturing chamber. 8-well coverglass (Sarstedt; 94.6170.802, left) were used to perform live imaging of human Langerhans islets (transmitted light microscopy, right).

Age (y)	Sex (M/F)	Diabetes (ND/T2D)	BMI (Kg/m2)	Cause of death	Stimulation index normalised for insulin content (a.u.)
**85**	**M**	**ND**	**27.7**	**cardiovascular disease**	**1.37**
88	M	ND	24.5	cardiovascular disease	1.47
52	F	ND	25.71	cardiovascular disease	1.87
81	F	ND	24.98	cardiovascular disease	2.04
**80**	**M**	**ND**	**23.03**	**cardiovascular disease**	**2.32**
82	F	ND	22.04	cardiovascular disease	2.82
72	F	ND	22.86	cardiovascular disease	3.01
81	M	ND	27.68	cardiovascular disease	3.16
**46**	**M**	**ND**	**23.67**	**trauma**	**3.9**
49	F	ND	31.22	cardiovascular disease	3.91
85	F	ND	23.44	cardiovascular disease	4.29
89	F	ND	19.53	cardiovascular disease	4.37
86	F	ND	27.05	cardiovascular disease	4.45
77	M	ND	26.2	cardiovascular disease	4.58
62	M	ND	37.04	cardiovascular disease	4.59
61	M	ND	24.8	trauma	4.78
**79**	**M**	**ND**	**26.81**	**cardiovascular disease**	**5.35**

Table S1: Donor-related personal data and insulin secretion assay results. Metabolic-imaged donors are reported in bold. Glossary: y: years, M/F: Male/Female, ND: Non diabetic, T2D: type 2 diabetes, BMI=Body Mass Index.

	**Mean α-cells Lipofuscin area (%)**	**Mean β-cells Lipofuscin area (%)**	**β/α ratio**	**Stimulation index normalised for insulin content (a.u.)**
**Donor 1**	10	18	1.71	1.37
**Donor 2**	7	14	2.11	2.32
**Donor 3**	3	7	2.02	3.9
**Donor 4**	8	15	1.95	5.35

Table S2: Donor lipofuscin quantity and β/α ratio in terms of lipofuscin quantity. increasing Mean α- and β-cell lipofuscin area values for each donor are obtained by averaging all the single-cell ratios between the lipofuscin-enriched area total cell area. β/α ratio is obtained by dividing the mean β-cell lipofuscin area by the mean α-cell lipofuscin area.

	β (%)	α (%)
**Islet 1**	92	8
**Islet 2**	78	23
**Islet 3**	58	42
**Islet 4**	63	37
**Islet 5**	64	36
**Islet 6**	78	22
**Islet 7**	44	56
**Islet 8**	64	36
**Islet 9**	53	47
**Islet 10**	57	43
**Islet 11**	53	47
**Islet 12**	63	37
**Islet 13**	92	8
**Islet 14**	83	17
**Islet 15**	77	23

Table S3: α/β cell proportions in all measured islets.

**Reagents**	**Company**	**Code**
2,2-Thiodiethanol	Merck	166782
Agarose	Merck	A9414
Calcium chloride	Merck	1.02391
D-Glucose	Gibco	15023021
Hepes	Thermo Fisher Scientific	11344041
Hoechst 33342	Thermo Fisher Scientific	H3570
Phosphate Buffered Saline	Thermo Fisher Scientific	18912
Potassium chloride	Avantor	0509
Potassium phosphate monobasic	Merck	P5655
Sodium chloride	Merck	71376
Triton X-100	Merck	T9284
**Primary Antibodies**	**Company**	**Code**
Anti-Glucagon	Merck	SAB4501137
Anti-Insulin	Bio-Rad	MCA1911G
**Secondary Antibodies**	**Company**	**Code**
Donkey Anti-Mouse Alexa Fluor 488	Thermo Fisher Scientific	A21202
Donkey Anti-Rabbit Alexa Fluor 594	Thermo Fisher Scientific	A21207

Table S4: Reagents utilized to perform the experiments.
